# Exosomal Gene Biomarkers in Osteosarcoma: Mifepristone as a Targeted Therapeutic Revealed by Multi‐Omics Analysis

**DOI:** 10.1096/fj.202501151RR

**Published:** 2025-07-05

**Authors:** Zheng Li, Jie Guo, Shaopeng Zhu, Yunpeng Zou, Wenqi Ma, Jiayao Niu, Ronghan Liu, Kai Zhao

**Affiliations:** ^1^ Central Hospital Affiliated to Shandong First Medical University Shandong First Medical University & Shandong Academy of Medical Sciences Jinan China; ^2^ School of Clinical Medicine Shandong Second Medical University Weifang China; ^3^ Bone Biomechanics and Metabolism Laboratory Central Hospital Affiliated to Shandong First Medical University Jinan China; ^4^ Spinal Surgery Department Central Hospital Affiliated to Shandong First Medical University Jinan China

**Keywords:** exosome, immune infiltration, machine learning, molecular docking, osteosarcoma

## Abstract

Osteosarcoma (OS) is an aggressive bone cancer that mainly occurs in children and adolescents. OS patients are mainly treated with neoadjuvant chemotherapy and surgical resection. This treatment is effective for early osteosarcoma. However, the effect declines as the disease progresses. Currently, our research on osteosarcoma is not enough to meet the clinical needs. Exosomes play a critical role in osteosarcoma progression by mediating intercellular communication. They carry molecular signals, including miRNAs and proteins, which can influence tumor growth, metastasis, and drug resistance. Recent studies have shown that exosomes from osteosarcoma cells can promote cell proliferation and migration, making them potential biomarkers for early diagnosis and therapeutic targets in osteosarcoma. This opens up new possibilities for the research of osteosarcoma. The combined genes of exosomes and DEGs were identified by searching GeneCards and GEO databases. Subsequent analyses included GO and KEGG Enrichment, GSEA. The core gene set was derived from the intersection of LASSO and SVM‐RFE outputs, ensuring minimal redundancy through dimensionality reduction. Osteosarcoma was diagnosed and predicted by differential expression levels, ROC curve analysis, and nomogram. Immune cell infiltration in osteosarcoma was evaluated by the ssGSEA algorithm. Drug enrichment analysis and molecular docking simulations were conducted to discover the most promising drug leads. In vitro experiments included Wound Healing Assay and qRT‐PCR to detect the therapeutic effect of the drug. Through multiple analyses and dimensionality reduction of the data set, six genes were selected (WNT5A, GCA, ANXA6, BIRC5, IL1β, and ARPC3). We examined differential expression in the control and tumor groups and made a gene prediction nomogram. Analysis of immune cell infiltration revealed significant alterations in the composition of immune cell subsets. Drug enrichment analysis and molecular docking of these six core genes were conducted to screen out the most suitable candidate drug: Mifepristone. Finally, Mifepristone was proved to inhibit the growth of osteosarcoma cells in vitro. Bioinformatics analysis identified six exosome‐associated osteosarcoma genes (WNT5A, GCA, ANXA6, BIRC5, IL1β, and ARPC3) that could serve as potential biomarkers. Through screening, Mifepristone, which can act on BIRC5 and IL1β at the same time, has a very effective osteosarcoma treatment effect.

## Graphical abstract

Integrated osteosarcoma and exosome gene datasets identified differentially expressed and module genes, with their intersection as candidate genes. Core genes were identified through enrichment analysis and machine learning. Core gene expression was compared between control and osteosarcoma groups. Immune infiltration revealed tumor microenvironment alterations. Drug enrichment analysis and molecular docking reveal that Mifepristone interacts with key genes (BIRC5 and IL1β). In vitro experiments demonstrate that Mifepristone effectively inhibits osteosarcoma cell growth and migration, suggesting its potential as a new treatment option.

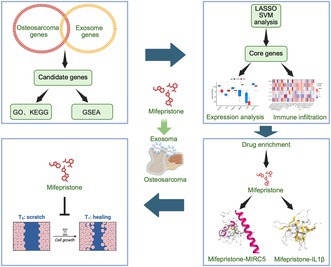

**Abbreviations**
AUC: area under the curve
BH: Benjamini‐Hochberg
BMMSCs: bone marrow mesenchymal stem cells
BP: biological process
CC: cellular component
DEG: differentially expressed gene
FDR: false discovery rate
GEO: gene expression synthesis
GO: gene ontology
GSEA: gene set enrichment analysis
HOB: human primary osteoblasts
KEGG: kyoto encyclopedia of genes and genomes
LASSO: least absolute shrinkage and selector operation
MET: mesenchymal to epithelial transition factor
MF: molecular function
NES: normalized enrichment scores
OS: osteosarcoma
PCA: principal component analysis
qRT‐PCR: quantitative real‐time PCR
ROC: receiver operating characteristic
ssGSEA: single‐sample gene‐set enrichment analysis
SVM‐RFE: support vector machine‐recursive feature elimination

## 1 Introduction

Osteosarcoma (OS) is a malignant osseous neoplasm originating from undifferentiated mesenchymal progenitor cells [1, 2]. Among all primary malignancies of the skeleton, osteosarcoma exhibits the greatest frequency of occurrence and mainly occurs in children and adolescents. Epidemiological data show that the incidence is about 4.8 per million, accounting for 0.2% of all malignancies [3, 4, 5]. It ranks third among the most common cancers in children under 20 years old [6, 7]. According to statistics, about 2% of tumors in children under 14 years of age and about 3% of tumors in children 14–19 years of age are osteosarcoma [8, 9]. A variety of risk factors have been reported for osteosarcoma, including increased birth weight, tall stature, specific hereditary cancer predisposition syndromes, and frequent genetic polymorphisms [10, 11]. Currently, OS patients are mainly treated with neoadjuvant chemotherapy and surgical resection. First‐line chemotherapeutic agents include adriamycin, cisplatin, and methotrexate [12]. This treatment is effective for early osteosarcoma. However, the effect declines as the disease progresses. Patients with advanced metastatic OS are often resistant to these chemotherapeutic agents, and few other effective targeted agents are available as second‐line treatment options, leading to an extremely poor prognosis for patients with advanced OS [13]. There are also many side effects, such as fluctuating systemic drug exposure, excessive clearance, non‐specific tissue distribution, cytotoxic effects on healthy cells, and prolonged use producing resistance. Therefore, their use in patients with osteosarcoma is limited [14]. In addition, the effects of osteosarcoma can be catastrophic, greatly impairing the holistic well‐being of patients and their family members, encompassing both somatic and psychological dimensions [15]. Thus, elucidating the molecular pathways underlying osteosarcoma advancement and pinpointing actionable therapeutic targets are urgent priorities, with the ultimate goal of designing targeted treatment modalities that demonstrate robust clinical efficacy.

Exosomes, a class of extracellular vesicles (30–150 nm) derived from the endosomal pathway, are released by diverse cell types, including cancer cells, stem cells, and immune cells [16, 17, 18, 19, 20]. Exosomes are not only involved in maintaining normal metabolism but also participate in regulating gene expression, immune responses, and pathological processes like tumor progression [21]. Exosomes mediate intercellular transfer of bioactive signals, triggering oncogenic pathways that drive proliferation, angiogenesis, and immunosuppression while evading antitumor immunity [22, 23]. Exosomes have the capacity to reprogram genetic transcription in target cells, thereby promoting multidrug resistance mechanisms, the process accomplished through the delivery of signaling molecules such as miRNA, proteins, and transcription factors [24]. At present, researchers believe that exosomes are promising in treating osteosarcoma [25]. Exosomes contribute significantly to unraveling osteosarcoma pathogenesis. Key exosomal molecules, including lncRNAs (PVT1 and XIST), miR‐21–5p, and CircNRIP1 secreted by bone marrow mesenchymal stem cells (BMMSCs) have been linked to tumor development [26, 27, 28, 29]. Macrophage‐secreted lncRNAs (Livr‐AS1 and OIP5‐AS1) and miR‐221–3p, together with tumor‐derived exosomal CTCF, drive osteosarcoma progression [30, 31, 32, 33]. Exosomes may increase osteosarcoma metastasis by regulating MMP or other processes, making prognosis worse [33]. Exosomes also change drug resistance in osteosarcoma. For example, luteolin‐primed exosomes restore adriamycin sensitivity in resistant osteosarcoma cells via miR‐384‐mediated inhibition of the PTN/β‐catenin/MDR1 pathway, offering a novel approach to combat chemoresistance [34]. Exosomes serve as critical mediators of intercellular signaling, providing valuable diagnostic biomarkers and therapeutic targets for osteosarcoma management [19]. Exosomes from osteosarcoma cells have been shown to carry miRNAs and proteins that regulate tumor growth, cell migration, and chemoresistance. Thus, exosomes, as key mediators of osteosarcoma progression, offer dual potential as biomarker sources and therapeutic targets, necessitating deeper exploration of their mechanisms.

**FIGURE 1:**
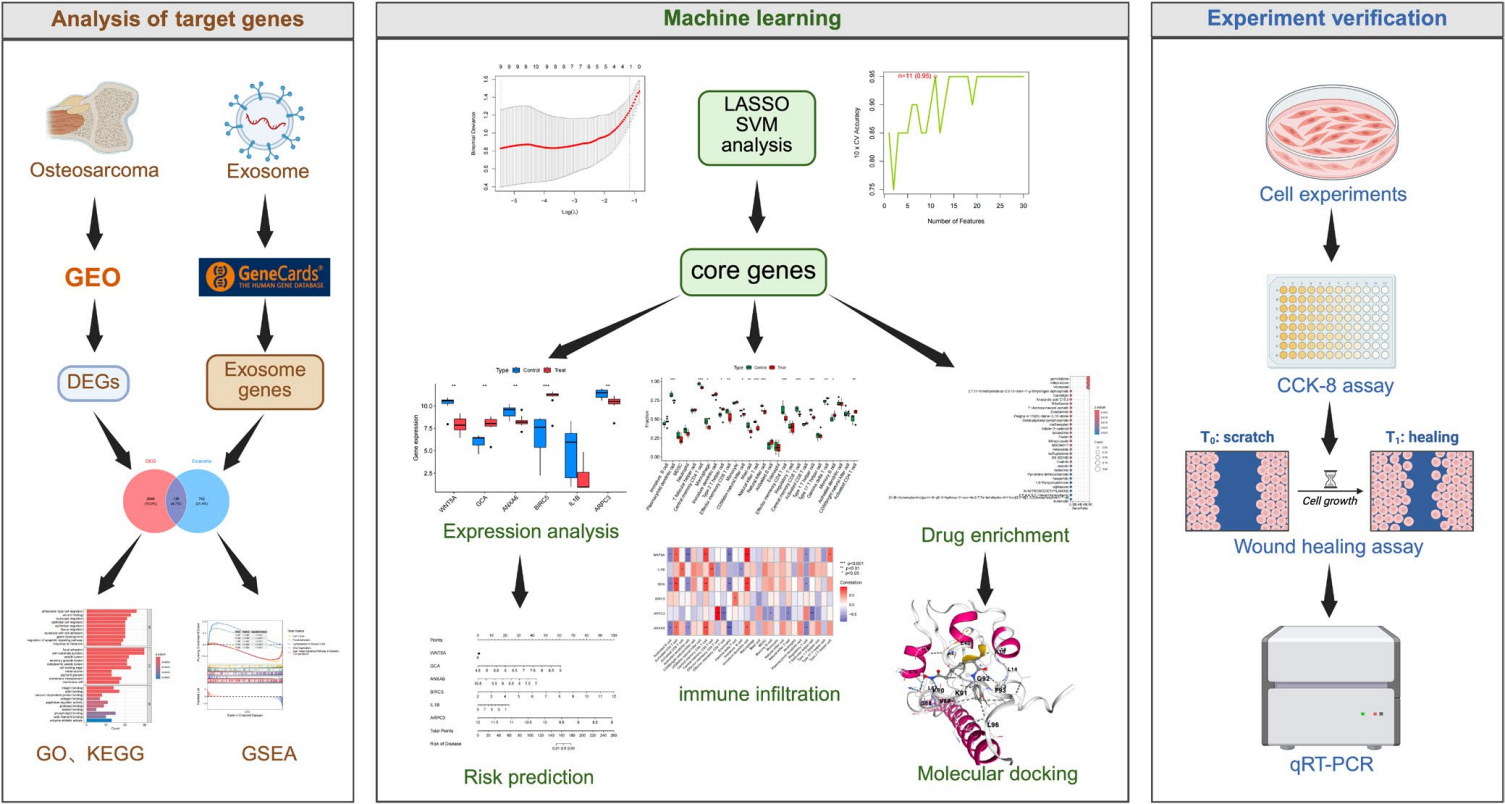
The workflow diagram of this study.

However, the study of exosome‐related genes in the osteosarcoma tumor environment is still not sufficient. The aim of this study was to identify exosome‐associated biomarkers in osteosarcoma and to evaluate the potential of drugs as a therapeutic agents by analyzing their effects on exosome‐mediated gene expression. During this research, we analyzed the expression of exosome‐related genes in osteosarcoma and established a machine learning‐based model for optimal prognosis and diagnostic prediction. In addition, R language was used for clinico‐pathological association studies, quantitative immunophenotyping, enrichment analysis, and chemotherapy drug sensitivity analysis of related genes. After screening, we found three drugs that work on three targets at the same time. In view of the intersection of the action targets of these three drugs and molecular docking, we screened out the final candidate drug with high binding energy: Mifepristone. The good therapeutic effect was verified by in vitro experiments. It can be used as a new and effective choice of clinical drugs and also provide a new insight for the management of osteosarcoma patients. The workflow is illustrated in Figure 1.

## 2 Materials and Methods

### 2.1 Data Source

Open‐access biomedical investigations and biospecimens fulfilling the specified inclusion criteria were searched in the National Center for Biotechnology Information's Gene Expression Repository (https://www.ncbi.nlm.nih.gov/geo/) database: (1) Expression data from human mesenchymal stem cells and osteosarcoma cells, (2) Expression profiles of human primary osteoblasts (HOB) and mesenchymal to epithelial transition factor (MET) transformed osteoblast clones. Two gene expression files (GSE28256, GSE30807) were identified and collected for further analysis.

Exosomes are nanoscale vesicles secreted by cells that mediate intercellular communication. These vesicles are involved in transporting proteins, lipids, and nucleic acids, which contribute to various biological processes, including cancer progression. Genes linked to exosomes were identified using GeneCards, with the keyword “Exosome” and filtered for “Protein Coding” and “Score > 2.” This selection prioritized genes involved in exosome biogenesis, ensuring relevance to endosomally derived vesicles. (https://www.genecards.org/) [35]. We detected 878 genes associated with exosomes.

### 2.2 Validation of the Gene Expression in Osteosarcoma‐Exosome Interactome

To characterize osteosarcoma‐exosome axis gene expression, we validated key transcripts in GSE28256 and GSE30807 cohorts. Principal component analysis (PCA) was employed to visualize group‐wise clustering patterns and assess multidimensional data distribution. Statistical significance was defined as *p* < 0.05 for all analyses.

### 2.3 Identification of Osteosarcoma‐Associated Genes Exhibiting Aberrant Expression Patterns

Identification of differentially expressed genes (DEGs) between normal and osteosarcoma tissues in the GSE28256 and GSE30807 datasets using the “limma” R package (v3.44.3) [36]. Differential expression thresholds (|log_2_FC| > 0.5, FDR < 0.05) were applied for visualization using ggplot2 (v3.3.2) and heatmap R packages (v0.7.7). These genes were crossed with 878 exosome‐associated genes to further obtain target genes. The results are presented in a Venn diagram.

### 2.4 Functional and Pathway Enrichment Analysis

Gene Ontology (GO) enrichment analysis was conducted across three primary categories: Molecular Function (MF), Cellular Component (CC), and Biological Process (BP) [37]. The KEGG database, a globally recognized resource, offers extensive insights into genetic material, physiological pathways, pathological states, and therapeutic compounds [38]. Functional enrichment analysis (GO/KEGG) was conducted on the DEG list using the clusterProfiler R package (v3.16.1) [39].

Corrected *p*‐values (*p*.adj) < 0.05 and false discovery rate (FDR) values (*Q* value) < 0.25 were considered statistically significant, and *p*‐values were corrected using the Benjamini‐Hochberg (BH) method to minimize false positives.

### 2.5 GSEA

For in‐depth characterization of osteosarcoma‐ and exosome‐related gene pathways, GSEA was performed using the clusterProfiler R package (v3.16.1) [39]. Pairwise correlations between key genes and all other genes in the training cohort were computed, followed by ranking of genes based on descending correlation coefficients to form the test set. The threshold was set to |NES| > 1, *p* value < 0.05.

### 2.6 Machine Learning‐Driven Identification of Candidate Genes

The least absolute shrinkage and selection operation (LASSO) and support vector machine recursive feature elimination (SVM‐RFE) algorithms were applied to identify candidate diagnostic genes in GSE28256 and GSE30807 datasets [40]. By applying the LASSO and SVM‐RFE algorithms for dimensionality reduction, redundant information can be minimized. The intersection of genes identified by both algorithms was defined as the core gene set. The results were also represented by a Venn diagram.

### 2.7 Expression Analysis and Diagnostic Prediction of Candidate Biomarkers

We utilized the “ggplot2” package to evaluate the expression levels of core‐related genes between the control and osteosarcoma groups in the GSE28256, GSE30807, and Exosome GeneCards datasets and performed *t*‐test to analyze differences (*p* < 0.05). Diagnostic performance of candidate biomarkers was evaluated using the “pROC” package to generate Receiver Operating Characteristic (ROC) curves, with AUC > 0.7 set as the optimal threshold. Furthermore, a multivariable logistic regression model was constructed to create a polygenic diagnostic classifier, with ROC curve analysis used to evaluate its predictive performance.

### 2.8 Establishment of a Nomogram Scoring System

We developed a predictive nomogram combining expression levels of core related genes and OS using the “rms” package in R. Each gene matched a score, and the scores of the six core genes for each sample were summed to obtain a total score. Construct the nomogram to predict disease risk by total score.

### 2.9 Single‐Sample Gene‐Set Enrichment Analysis (ssGSEA)

To quantify the proportional abundance of tumor‐infiltrating immune cells, the ssGSEA [41] methodology was used. The ssGSEA algorithm in the R software package GSVA was used to calculate the enrichment fraction to determine the abundance of distinct immune cell subsets within the biological specimens. Box plots were generated to depict the differences in immune cell infiltration between the control and osteosarcoma groups in the dataset. Matrix plots were subsequently employed to investigate the association between dataset‐derived hub genes and immune cell landscapes. Visualization of the correlation data was achieved using the “ggplot2” library in R.

### 2.10 Molecular Docking

The crystal structures of compounds were obtained from the PubChem database (https://pubchem.ncbi.nlm.nih.gov)f and the proteins were obtained from the PDB database (https://www.rcsb.org). CB‐DOCK2 docking software (https://cadd.labshare.cn/cb‐dock2/php/index.php) was used for molecular docking of three candidate drugs Gemcitabine (PubChem CID:60750), Mifepristone (PubChem CID:55245) and Vorinostat (PubChem CID:5311) to the common targets BRIC5 (PDB ID:3UEC) and IL1β (PDB ID:LS0L) [42].

### 2.11 Cell Culture and Drug Treatment

Human U‐2 OS「U2OS」cell line was purchased from Procell Life Science & Technology Co. Ltd. U2OS cells were cultured in U2OS special medium (Procell). The cell line was cultured at 37°C in a humidified incubator containing 5% CO_2_.

Mifepristone (purity > 95%, #CAS 84371‐65‐3) was obtained from MCE (Shanghai, China) and dissolved in Dimethyl sulfoxide (Solarbio, Beijing, China).

### 2.12 CCK‐8

U2OS cells in the exponential growth period were harvested and cultured until the fusion degree reached 70%–80%. Then, these cells were exposed to a series of Mifepristone concentrations: 1 μM, 5 μM, 10 μM, 50 μM, and 100 μM, and then cultured for a period ranging from 1 to 2 days. Cell proliferation and cytotoxic reaction were quantified by using the CCK‐8 assay (Abclonal, Wuhan, China). The MultiskanGo full‐spectrum microplate reader is used at a wavelength of 450 nm.

### 2.13 Wound Healing Assay

U2OS cells were cultured in 6‐well plates. When the cells had grown to 100% density, lines were drawn in each hole with a sharp instrument to separate the cells, and photographs were taken under a microscope at 0 h. The cells were treated with 5 μmol/L or no Mifepristone. After waiting 24 h and 48 h, we filmed the migration of each well. Finally, the migration area between different time points is calculated using ImageJ software, and the mobility is obtained.

### 2.14 Quantitative Real‐Time PCR (qRT‐PCR)

Total RNA was extracted from human osteosarcoma U2OS cells with TRIzol reagent (Invitrogen), following the product instructions. cDNAs were generated and transcribed with the Evo M‐MLV RT Kit (Accurate Biology). Quantitative real‐time PCR analysis was conducted using the Roche LightCycler480 System with SYBR Green‐based detection chemistry (Accurate Biology qPCR Kit), following vendor‐recommended protocols. Ultimately, the 2^−ΔΔCt^ quantification algorithm was applied to determine the relative mRNA abundance. The primer sequence is shown in Table 1.

**TABLE 1:** PCR primer sequence.

Gene	Pre‐primer sequence	Post‐primer sequence
β‐Actin	TGACGTGGACATCCCCAAAG	CTCGAAGGTCGACACCCAGG
LACTB	GCCATGGCGATCTACGTG	CAGGTGCTGGTAGAGGTC
c‐Myc	TCCAGCTTGTACCTGCAGGATCTGA	TCAGACCTAGTGGAAGACGACCTCC
Mcl‐1	CGCCCGGGAGGGCGACTTTTG	TGGAAGAACTCCACAAACCCATC

### 2.15 Statistical Analysis

The dataset was computationally processed and statistically analyzed using the R software (version 4.2.2). An unpaired Student's *t*‐test was applied to compare quantitative measurements between the two experimental groups under the assumption of normal distribution. When data were not normally distributed to assess differences between groups, the Mann–Whitney *U*‐test was used. The Kruskal‐Wallis test was utilized for comparisons involving more than two groups.

Depending on the characteristics of the dataset, discrete categorical variables between the two study cohorts were evaluated using either Pearson's *χ* ^2^ test or Fisher's exact probability test. Spearman's rank correlation test was conducted to investigate the associations between variables. Two‐tailed *p*‐values were computed for all analyses, with statistical significance defined as *p* < 0.05.

## 3 Results

### 3.1 Identification of OS‐Related Target Genes

**FIGURE 2:**
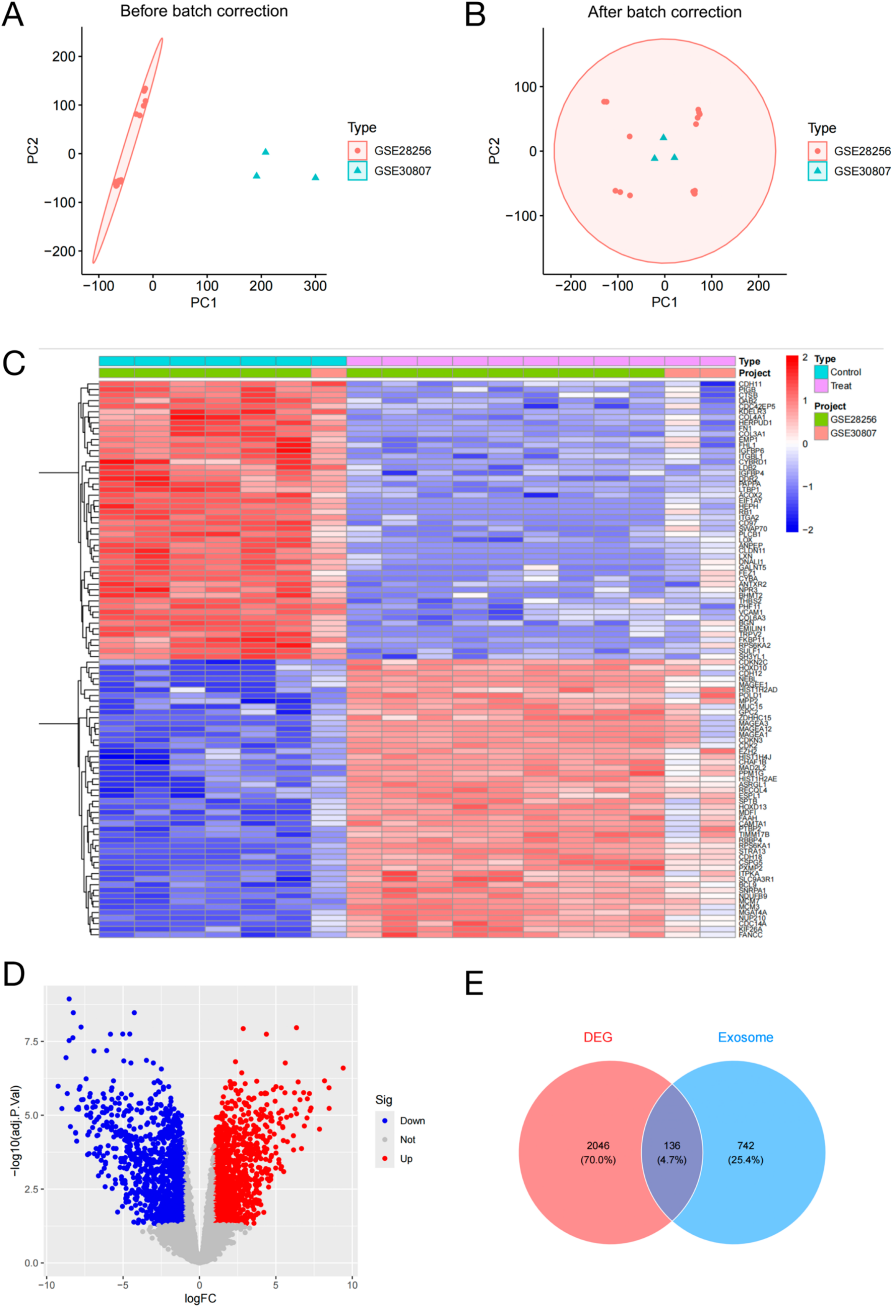
Data collection and consolidation. (A) Data before batch effect removal. (B) Data after batch effect removal. (C) Heat map of significantly different genes. (D) Volcano plot of significantly different genes. (E) Venn diagram of intersecting target genes for exosome and OS.

Expression profiles from datasets GSE28256 and GSE30807 were combined to elucidate genes associated with osteosarcoma pathology. A significant reduction in batch effect was shown by PCA and homogeneity between samples was achieved (Figure 2A,B).

### 3.2 Acquisition of Disease Targets Associated With Exosome

Differential expression analysis of integrated OS samples revealed DEGs. We created a heat map to visualize DEGs (Figure 2C). A volcano plot was also created to visualize significantly different genes (Figure 2D). Through integrative analysis of multi‐source databases and subsequent removal of redundant entries, a total of 2182 candidate protein targets were discovered. The overlap analysis between DEGs and exosome‐associated target genes identified 136 common targets, which were subsequently visualized using a Venn diagram representation (Figure 2E).

### 3.3 Enrichment Analysis of Intersecting Target Genes

**FIGURE 3:**
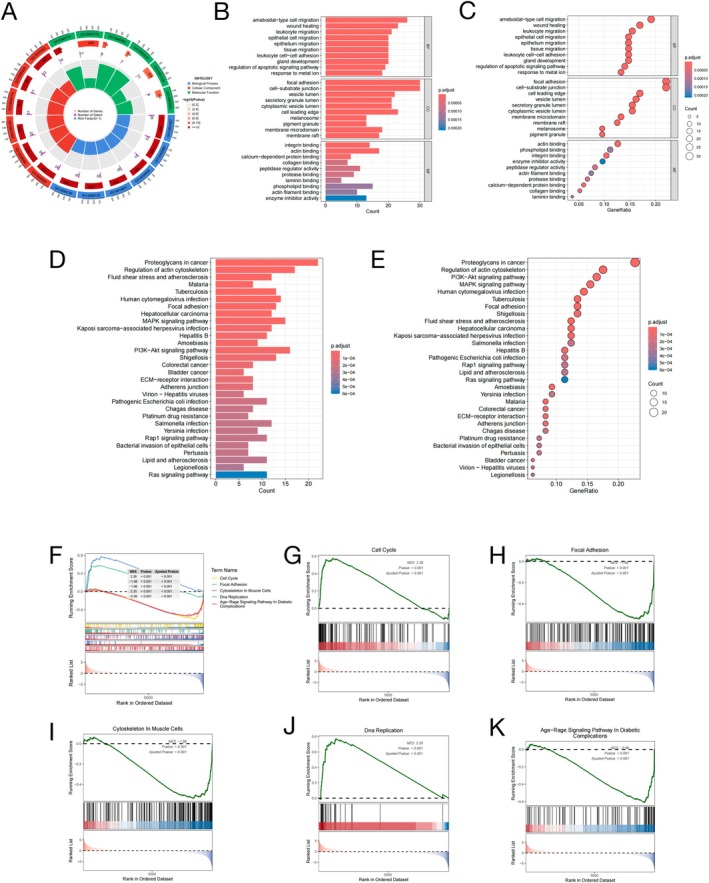
Enriched KEGG pathway, GO terms and gene set enrichment analysis (GSEA). (A) GO enrichment analysis and chord plot. (B) GO enrichment analysis and bar plot. (C) GO enrichment analysis and bubble plot. (D) KEGG enrichment analysis and bar plot. (E) KEGG enrichment analysis and bubble plot. (F–K) GO/KEGG enrichment analyses using GSEA for the intersecting gene (Top5).

The 136 identified genes are potential targets linked to osteosarcoma and exosome. Functional characterization was performed using GO term enrichment and KEGG pathway mapping to elucidate their biological roles. For the GO analysis of 136 potential targets, the focus was on humans ( *Homo sapiens* ) as the study species. A total of 343 statistically significant GO items were identified through the analysis, including 130 BP items, 147 CC items, and 66 MF items (Figure 3A). The top 10 GO terms per BP/CC/MF category, ranked by FDR‐adjusted significance, were visualized in the enrichment plot (Figure 3B,C). Furthermore, pathway enrichment analysis was conducted on the 136 candidate targets through DAVID and FUMA platforms to elucidate their involvement in specific signaling cascades. Application of an FDR < 0.05 significance threshold enabled the prioritization of 94 target‐associated signaling pathways from the pathway enrichment analysis. The 30 most prominent pathways were visualized through integrated histogram and bubble chart representations to enhance data interpretation (Figure 3D,E).

### 3.4 GSEA Enrichment Analysis

Relationships between gene expression and relevant biological processes, cellular components, and molecular functions in the dataset are investigated through GSEA. Pathway overrepresentation analysis was conducted to detect biologically meaningful enrichments, applying dual significance thresholds of adjusted *p* < 0.05 and *q* < 0.25 to control false discovery rates. The five pathways demonstrating the most significant normalized enrichment scores (NES) were prioritized for display, with results graphically represented using GSEA's classical enrichment map (Figure 3F). The analysis identified marked overrepresentation of genes in biological processes including: Cell Cycle (Figure 3G), Focal Adhesion (Figure 3H), Cytoskeleton in Muscle Cells (Figure 3I), DNA Replication (Figure 3J), and Aged‐Range Signaling Pathway in Diabetic Complications (Figure 3K).

### 3.5 Machine Learning Identification of Exosome‐Related DEGs

**FIGURE 4:**
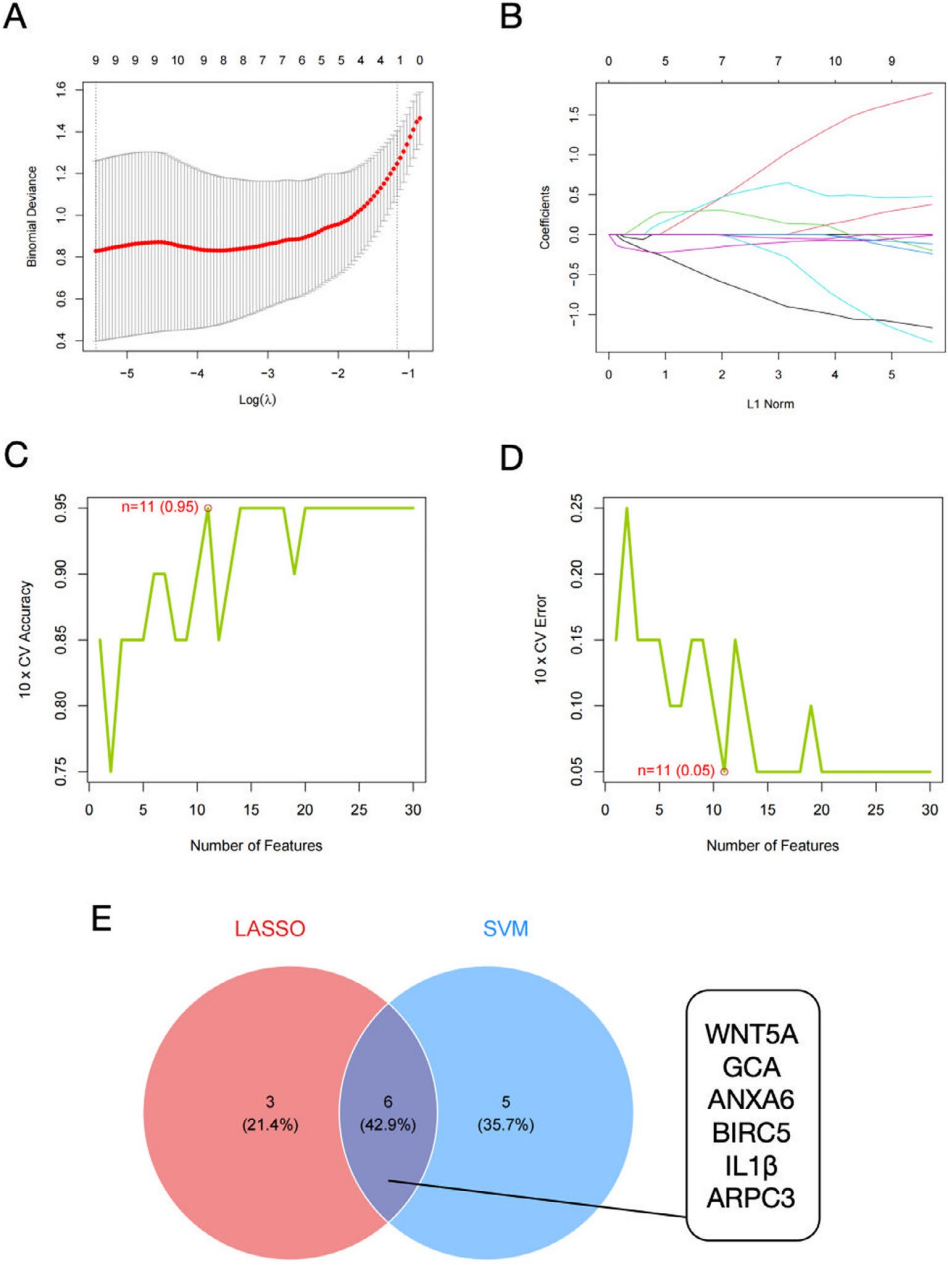
Targeted genes screening through LASSO regression and SVM‐RFE analyses. (A) Error plot for the logistic LASSO regression coefficient. (B) Nine genes were screened in the logistic LASSO model. (C) Plot of generalization accuracy versus the number of features. (D) Plot of generalization error versus the number of features. (E) Venn diagram of six intersection genes.

We successfully utilized lasso regression to screen for important exosome‐related DEGs, produced gene error plots (Figure 4A) and coefficient plots (Figure 4B) for cross‐validation. Nine differentially expressed genes were further characterized by using SVM‐RFE. We plotted a graph of generalization accuracy against the number of features and employed 10‐fold cross‐validation to select the eigengenes (Figure 4C). We plotted a graph of generalization error against the number of features and utilized 10‐fold cross‐validation to identify and select the eigengenes (Figure 4D). The intersection of gene features selected through LASSO and SVM‐RFE identified six consensus core genes (WNT5A, GCA, ANXA6, BIRC5, IL1β, and ARPC3) as displayed in the Venn diagram (Figure 4E).

### 3.6 Differential Expression Analysis and ROC Analysis of Osteosarcoma

**FIGURE 5:**
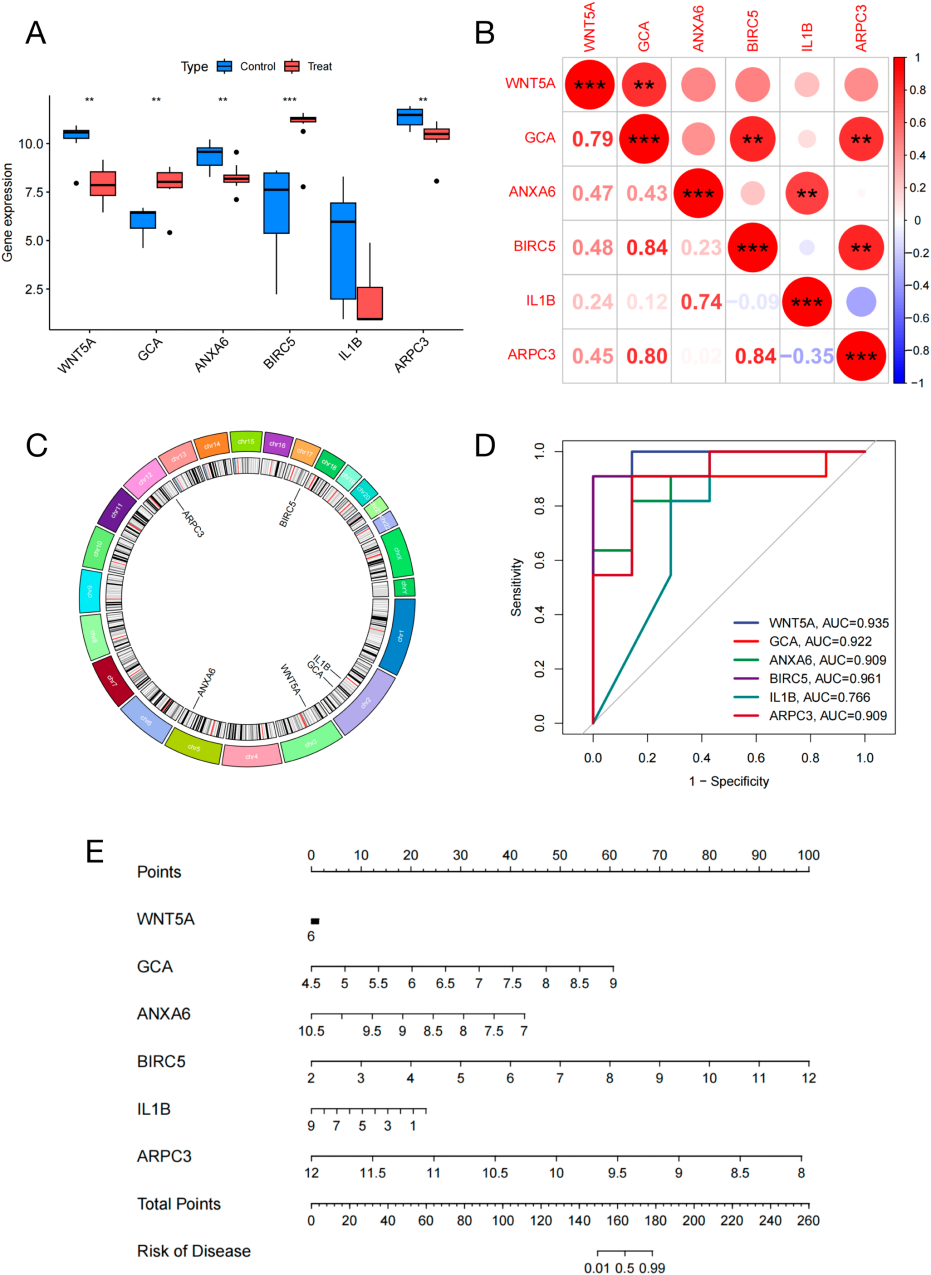
The expression of the six prognostic OS‐related genes. (A) The expression levels of these six genes in the control and osteosarcoma groups. (B) The correlation among the six genes expression. (C) Chromosomal distribution of the OS‐DEGs visualized in a circular format. (D) The time‐dependent ROC curves. (E) The nomogram combining total scores of different gene expression levels was developed to retrieve the corresponding risk prediction.

The expression profiles of six core genes (WNT5A, GCA, ANXA6, BIRC5, IL1β, and ARPC3) were contrasted between the control and osteosarcoma groups. Findings from the differential expression study are visualized in the between‐group comparison diagram (Figure 5A). Compared with the normal group, GCA, ANXA6, and BIRC5 in the cancer group were increased. The IL1β, ARPC3, and WNT5A decreased. The correlation information of these genes is presented (Figure 5B). These findings emphasized notable differences in the expression of the six genes between the control group and the osteosarcoma group (*p* < 0.05). To examine the chromosomal locations of these six genes, annotations were created utilizing the RCircos package (Figure 5C). Furthermore, ROC analysis confirmed their strong diagnostic potential for osteosarcoma (Figure 5D). The findings revealed that BIRC5 exhibited a high diagnostic accuracy for OS, with an AUC of 0.961. Other genes, including WNT5A (AUC = 0.935), GCA (AUC = 0.922), ANXA6 (AUC = 0.909), ARPC3 (AUC = 0.909), and IL1β (AUC = 0.766) also exhibited heterogeneous levels of predictive precision for detecting osteosarcoma. Based on the above regression analysis, we developed a nomogram that provides a quantitative prediction method for clinicians. A score is obtained for each gene expression level for each patient, and the resulting total score can be used to retrieve the corresponding risk prediction. When the total score is between 176 and 180, there is a very high probability that osteosarcoma can be diagnosed (Figure 5E).

### 3.7 Estimation of Tumor Immune Microenvironment and Immune‐Related Genes

**FIGURE 6:**
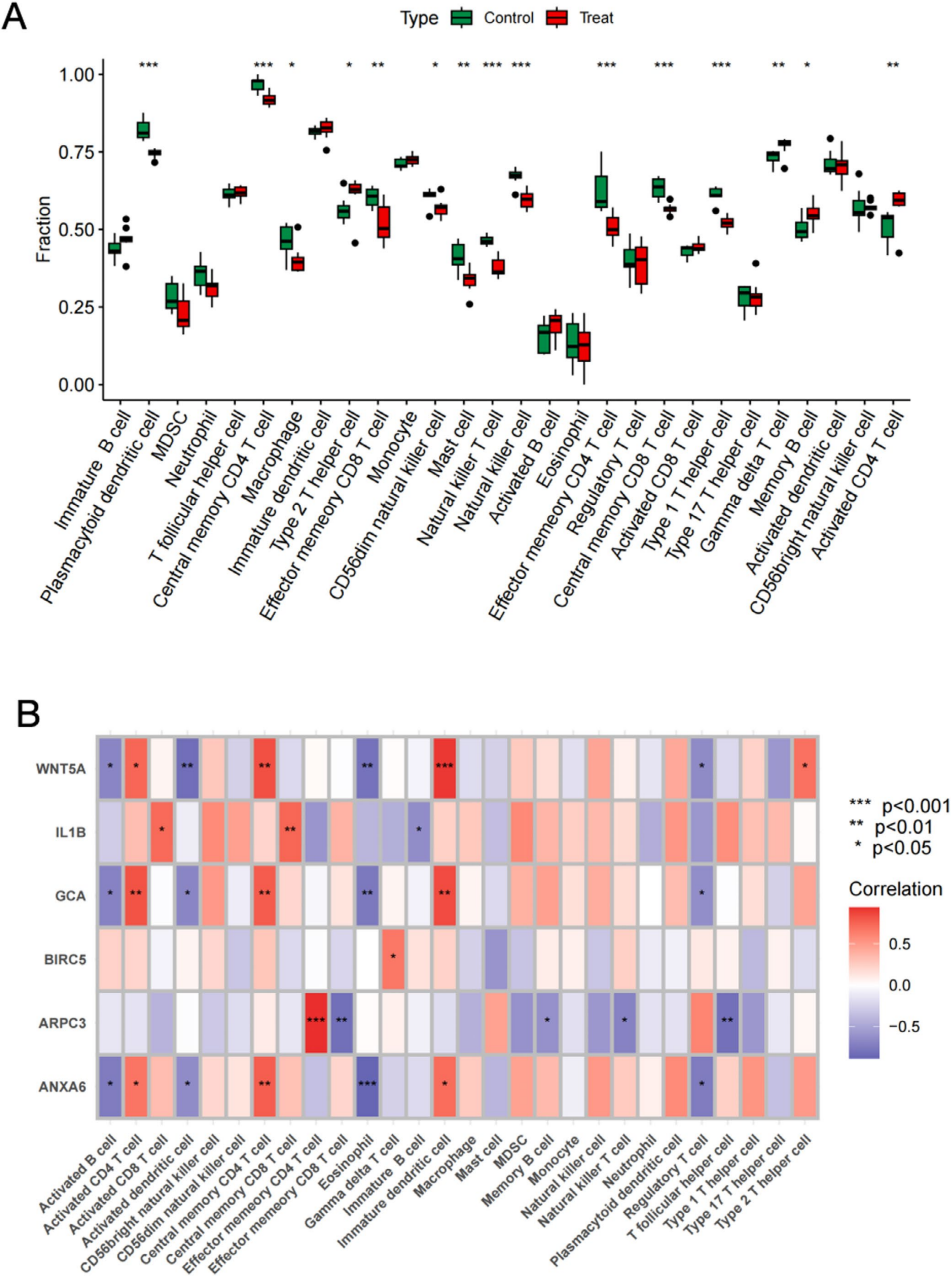
Analysis of tumor immune microenvironment and immune infiltration. (A) Box plot of grouping comparison of immune cells normal and tumor groups. (B) Heat map of correlations between the 28 immune cells.

In order to determine the variations in infiltrating immune cells between the normal and tumor groups across 28 distinct immune cell types, the ssGSEA computational framework was employed to characterize dominant cellular populations. Visualize the results using group comparison boxplots (Figure 6A). Significant disparities (*p* < 0.05) were detected in the abundance profiles of diverse immune cell subsets when comparing the normal and tumor groups. Further investigation explored the correlation between the infiltration patterns of 28 distinct immune cell populations in osteosarcoma biopsies and the transcriptional activity of six targeted genes. This correlation was visually represented through a heat map (Figure 6B).

### 3.8 Drug Enrichment Screening of Potential Therapeutic Drugs

**FIGURE 7:**
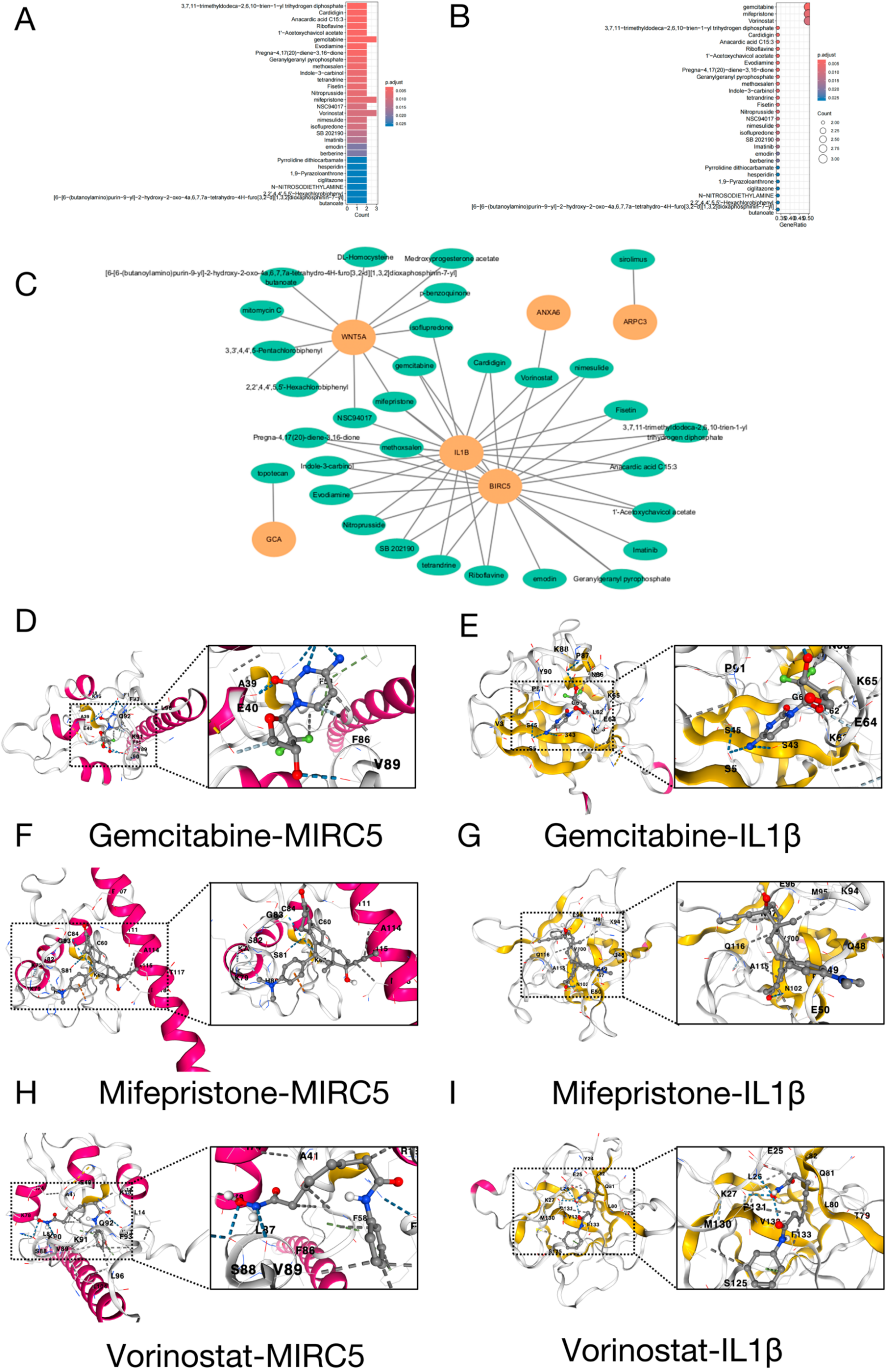
Effective binding of drugs to target proteins. (A) Drug enrichment analysis and bar plot. (B) Drug enrichment analysis and bublle pot. (C) Interaction network of drugs‐targets. (D–I) Docking results of core targets and molecules.

To predict drugs interacting with the six genes (WNT5A, GCA, ANXA6, BIRC5, IL1β, and ARPC3), we looked at PubChem for drugs that target these six genes. We conducted enrichment analysis of the drugs and selected the top 30 drugs with *p*‐value (Figure 7A). Then, according to the number of bindings between the drug and the target gene, the top three candidate drugs that bind to three targets were selected (Figure 7B). These were Gemcitabine, Mifepristone, and Vorinostat.

The results of drug enrichment and six key genes were introduced into Cytoscape ver 3.9. The data was combined into a network of drug‐target (Figure 7C). The protein is depicted as an oval with a pale orange hue, while the drug is prominently highlighted as a green oval shape. According to the drug target network cluster, we found the common target of three drugs: BRIC5 and IL1β.

### 3.9 Molecular Docking of Three Candidate Drugs to the Common Targets

Molecular docking is widely used to study potential binding patterns between molecules. This investigation utilized molecular docking simulations to characterize the most favorable binding conformation of three candidate drugs to their common targets. The simulation outputs elucidate critical parameters including affinity scores, interfacial amino acid contacts, and non‐bonded interaction profiles. Two core targets were selected: BRIC5 and IL1β, and molecular docking simulations with three candidate drugs: Gemcitabine, Mifepristone, and Vorinostat were conducted using CB‐DOCK2 docking software [42] (Figure 7D–I).

All six simulation outputs demonstrated favorable affinity scores (binding affinity < −5), suggesting a robust interaction between the selected targets and the drugs (Table 2). Gemcitabine: BRIC5 (−6 kcal/mol) and IL1β (−5.9 kcal/mol). Mifepristone: BRIC5 (−6.6 kcal/mol) and IL1β (−6.7 kcal/mol). Vorinostat: BRIC5 (−6.6 kcal/mol) and IL1β (−5.9 kcal/mol). According to the docking score, Mifepristone has the best protein affinity.

**TABLE 2:** Details of the molecular docking information.

Name	(Kcal/mol)	Contact residues
Gemcitabine‐MIRC5	−6	PHE13 LYS15 ARG18 ALA39 GLU40 ALA41 GLY42 PHE58 ILE74 HIS77 LYS78 PHE86 LEU87 SER88 VAL89 LYS90 LYS91 GLN92 PHE93 GLU94 GLU95 LEU96
Gemcitabine‐IL1B	−5.9	VAL3 SER5 LEU6 ASN7 SER43 SER45 GLY61 LEU62 LYS63 GLU64 LYS65 ASN66 LEU67 TYR68 VAL85 ASP86 PRO87 LYS88 TYR90 PRO91 SER153
Mifepristone‐MIRC5	−6.6	CYS60 PHE61 LYS62 GLU63 LYS78 LYS79 HIS80 SER81 SER82 GLY83 CYS84 ALA85 GLU107 LYS110 ASN111 ALA114 LYS115 THR117 ASN118 ASN119 LYS121 LYS122
Mifepristone‐IL1B	−6.7	GLN48 GLY49 GLU50 LYS55 PRO57 LYS92 LYS94 MET95 GLU96 LYS97 ARG98 VAL100 ASN102 GLU113 SER114 ALA115 GLN116 PRO118 ASN119
Vorinostat‐MIRC5	−6.6	LEU6 PRO7 TRP10 PHE13 LEU14 LYS15 ASP16 HIS17 ARG18 GLU40 ALA41 PHE58 ILE74 LYS78 ALA85 PHE86 LEU87 SER88 VAL89 LYS90 LYS91 GLN92 PHE93 GLU94 GLU95 LEU96 THR97 LEU98 GLY99 GLU100 PHE101 LYS103 LEU104 GLU107
Vorinostat‐IL1B	−5.9	PRO23 TYR24 GLU25 LEU26 LYS27 LEU69 LYS74 ASP75 LYS77 PRO78 THR79 LEU80 GLN81 LEU82 GLU83 SER84 TRP120 SER125 MET130 PRO131 VAL132 PHE133 LEU134 GLY135

### 3.10 Therapeutic Effects of Mifepristone

**FIGURE 8:**
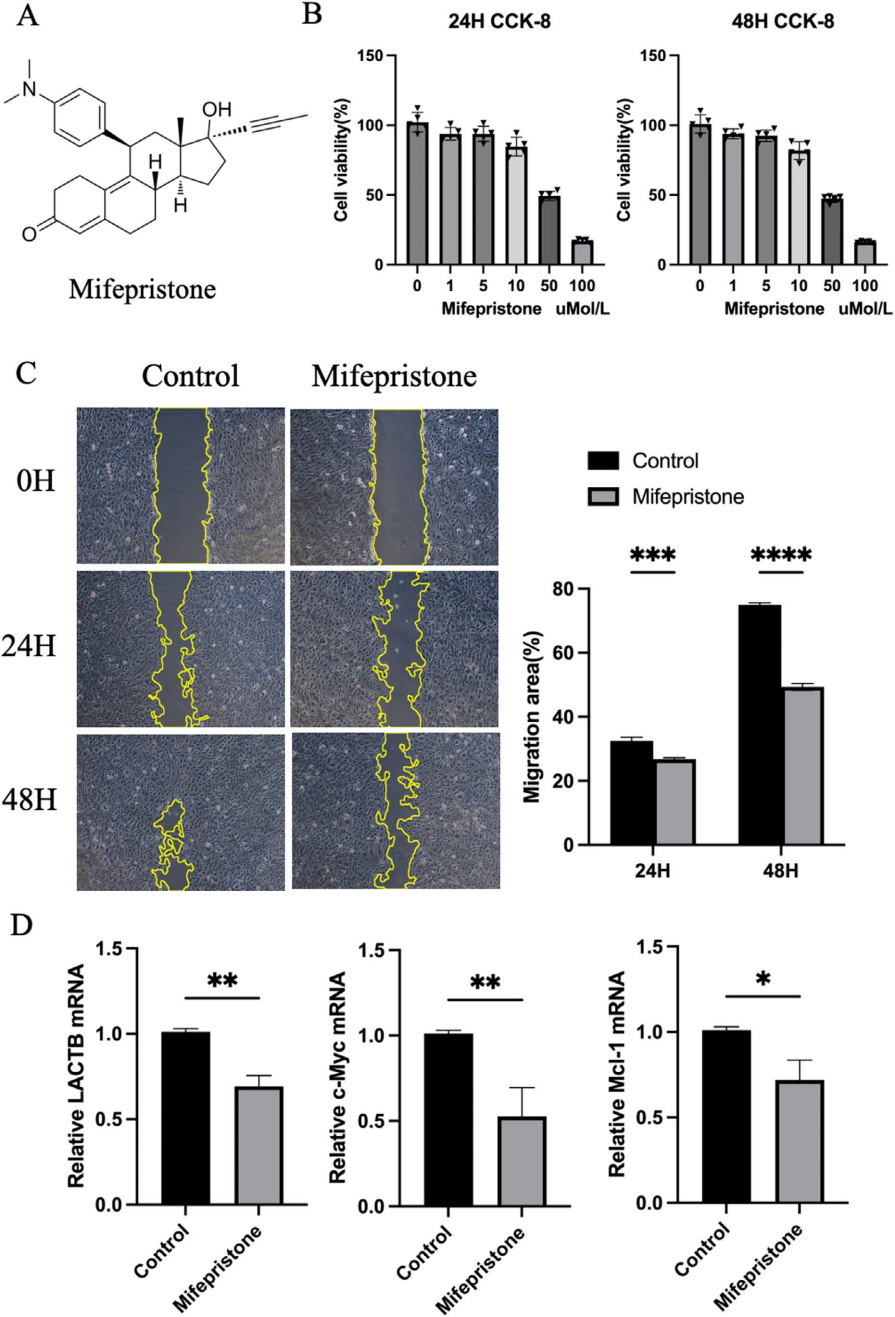
Therapeutic effects of mifepristone. (A) Chemical structure of Mifepristone, as the final candidate of the screened drugs. (B) 24‐ and 48‐ h CCK‐8 statistics of Mifepristone intervention in U2OS cell. (C) Wound healing assay was used to detect the migration rate. (D) PCR plots of pivotal genes of Mifepristone intervention in U2OS cell (NS statistically insignificant, *p < 0.05, **p < 0.01, and ***p < 0.001).

Given that the safety of Mifepristone was our primary concern, we had to design some tests to ensure its safety in cells. We selected the human osteosarcoma U2OS cell line and added Mifepristone at different doses (up to 100 μM) for 1 to 2 days (Figure 8A). There was no significant difference between 24 and 48 h in cell activity detected by the CCK‐8 assay. We analyzed the optimal time and dose of Mifepristone intervention in osteosarcoma cells at 5 μmol/L for 24 h (Figure 8B). The wound healing assay showed that Mifepristone led to a significant reduction in cell migration and invasion ability (Figure 8C). The expression of carcinogenic proteins LACTB, c‐Myc, and Mcl‐1 related RNA in the osteosarcoma U2OS cell line detected by qRT‐PCR could be inhibited by Mifepristone intervention (Figure 8D). The above experimental results preliminarily prove that Mifepristone can effectively suppress the growth of osteosarcoma cells and the expression of carcinogenic proteins, so as to play a role in the treatment of osteosarcoma.

## 4 Discussion

Osteosarcoma is a highly heterogeneous malignant tumor of bone origin that lacks effective therapeutic targets. Currently, OS patients are mainly treated with neoadjuvant chemotherapy and surgical resection. First‐line chemotherapeutic agents include adriamycin, cisplatin, and methotrexate [12]. However, patients with advanced metastatic OS are often resistant to these chemotherapeutic agents and few other effective targeted agents are available as second‐line treatment options, leading to an extremely poor prognosis for patients with advanced OS [13]. Exosomes are nanoscale extracellular vesicles wrapped in membranes released by different cells [16, 17]. Exosomes constitute a critical determinant of intercellular communication and signal molecule transfer, consequently triggering oncogenic signaling cascades that drive tumor growth, neovascularization, and escape from immune surveillance, while also inhibiting anti‐tumor immune responses [22, 23]. Currently, researchers believe that exosomes hold broad prospects in the treatment of osteosarcoma [25]. Exosomes have a significant role in the initiation and metastatic progression of osteosarcoma. However, the research on exosomes in osteosarcoma is limited and needs to be explored.

We first downloaded the GSE28256 and GSE30807 dataset and obtained 2182 DEGs using the filtering criteria FDR < 0.05 and |log2 Fiber Channel | > 0.5. By cross‐analysis of 878 exosome‐associated genes and 2182 DEGs, 136 exosome‐associated DEGs were identified. LASSO and SVM were used to analyze the dataset to further concentrate the number of genes, ultimately identifying 9 and 11 genes, respectively. At the intersection of the two analysis results, 6 shared genes were obtained (WNT5A, GCA, ANXA6, BIRC5, IL1β, and ARPC3). We examined their correlation to each other and their position on the chromosome. Subsequently, we quantified the transcriptional profiles of these six genes in normal and osteosarcoma tissues. Risk predictions were retrieved by combining the total scores of different gene expression levels.

To ascertain the differences in infiltrating immune cell populations between normal and osteosarcoma groups across 28 distinct immune cell types, we utilized the ssGSEA algorithm to identify the predominant cell types. Subsequent bioinformatic investigations explored the statistical association between the infiltration patterns of these 28 immune cell subsets and the transcriptional activity of six candidate genes. To predict drugs interacting with the six genes, we looked at PubChem for drugs that target these six genes. We conducted enrichment analysis of the drugs and selected the top 30 drugs with *p*‐value. We screened three drugs that bind to three targets: Gemcitabine, Mifepristone, and Vorinostat. The next step is to make molecular docking prediction based on the two common targets: BRIC5 and IL1β. According to the level of binding energy, Mifepristone, with the highest binding energy, was selected as the candidate drug.

Mifepristone is known to be an antiprogesterone with a high affinity for progesterone and glucocorticoid receptors. It has received attention for its receptor‐binding ability and potential to regulate hormonal responses [43]. It is a competitive antagonist of the progesterone receptor during pregnancy and exhibits its reversible binding [44]. However, studies of mifepristone in osteosarcoma are very limited. Exosomes play an important role in osteosarcoma. Emerging evidence demonstrates that exosome‐derived microRNAs (miRNAs) critically regulate multiple oncogenic processes, including cellular motility, growth, invasiveness, programmed cell death, and therapeutic resistance across diverse malignancies. Illustratively, luteolin‐treated sensitive cells release exosomes that enhance the chemosensitivity of adriamycin‐resistant cancer cells through miR‐384 upregulation and subsequent suppression of the PTN‐β‐catenin‐MDR1 signaling cascade. This approach constitutes a novel therapeutic strategy for overcoming chemotherapy resistance in osteosarcoma patients [34]. Therefore, we hypothesized that mifepristone could be used to treat osteosarcoma by targeting exosome genes. In order to achieve the role of new use of old drugs, we aim to reduce the economic burden of new drug development.

We found that the previously reported tumor suppressor proteins LACTB, c‐Myc, and Mcl‐1 were highly expressive in osteosarcoma and with a robust inverse correlation to patient survival outcomes [45, 46]. The appropriate concentration of mifepristone was selected by CCK‐8 assay. Mifepristone can significantly inhibit the cell proliferation of the human osteosarcoma U2OS cell line. Moreover, it can reduce the expression level of osteosarcoma‐associated tumor suppressor proteins. This indicates the potential of this drug for treating osteosarcoma. Our research found that compared to normal tissues, the expression level of MIRC5 was increased in osteosarcoma tissues, while the expression of IL‐1β was decreased. Given the abnormal expressions of these two genes, we inferred that MIRC5 might be a cancer‐promoting gene. On the contrary, IL‐1β acts as a tumor suppressor gene. They simultaneously or successively affect the proliferation, apoptosis, invasion, and metastasis abilities of osteosarcoma cells. Mifepristone, as a potential drug that targets both markers simultaneously, has been verified to have a certain inhibitory effect on osteosarcoma cell lines after preliminary exploration. Therefore, we infer that it may inhibit MIRC5 and promote the expression of IL‐1β to achieve the inhibitory effect on osteosarcoma. Thus, mifepristone treatment may lead to significant changes in the expression of genes related to exosome generation and function, achieving the desired therapeutic effect. However, the precise molecular mechanisms underlying this phenomenon require further elucidation through systematic investigation.

In short, we demonstrated the pivotal role of exosomes in the development of osteosarcoma through multiple analyses. Second, we identified promising genes related to exosomes for the treatment of osteosarcoma. Finally, based on these genes, we conducted drug screening and verified its therapeutic effect. It offers a fresh perspective for enhancing our comprehension of exosomes' role in the etiology, development, and management of osteosarcoma. This work focused on exosomes originating from the endosomal pathway; future studies should explore contributions from other EV subtypes (e.g., microvesicles or apoptotic bodies) to osteosarcoma biology.

## Author Contributions

Z.L., J.G., and S.Z. contributed to the study design and conceptual framework. Y.Z., W.M., and J.N. conducted data acquisition, performed statistical analysis, and interpreted the results. Technical support was provided by Z.L., J.G., S.Z., Y.Z., W.M., and J.N. Manuscript composition and critical revision were completed by Z.L., R.L., and K.Z. All authors have reviewed and approved the final version of the manuscript.

## Conflicts of Interest

The authors declare no conflicts of interest.

## Data Availability

The data achieved and analyzed in the current study are available in the GEO database (https://www.ncbi.nlm.nih.gov/geo/) and the GeneCards database (https://www.genecards.org/). Further reasonable inquiries can be directed to the corresponding author.
